# The lower incidence of cervical cancer in type 2 diabetes mellitus with sodium-glucose cotransporter 2 inhibitors utilization

**DOI:** 10.7150/jca.101165

**Published:** 2024-10-14

**Authors:** Tung-Lin Tsui, Yung-Chuan Ho, Kwo-Chang Ueng, Pei-Lun Liao, Jing-Yang Huang, Chia-Yi Lee, Shih-Chi Su, Shun-Fa Yang

**Affiliations:** 1 Institute of Medicine, Chung Shan Medical University, Taichung, Taiwan.; 2 Division of Cardiology, Department of Internal Medicine, Camillian Saint Mary's Hospital Luodong, Luodong, Yilan, Taiwan.; 3 Center for General Education, Chung Shan Medical University, Taichung, Taiwan.; 4 School of Medicine, Chung Shan Medical University, Taichung, Taiwan.; 5 Division of Cardiology, Department of Internal Medicine, Chung Shan Medical University Hospital, Taichung, Taiwan.; 6 Department of Medical Research, Chung Shan Medical University Hospital, Taichung, Taiwan.; 7 Department of Ophthalmology, Nobel Eye Institute, Taipei, Taiwan.; 8 Whole-Genome Research Core Laboratory of Human Diseases, Chang Gung Memorial Hospital, Keelung, Taiwan.; 9 Department of Medical Biotechnology and Laboratory Science, College of Medicine, Chang Gung University, Taoyuan, Taiwan.

**Keywords:** SGLT2 inhibitors, epidemiology, cervical cancer, age, type 2 diabetes mellitus

## Abstract

Sodium-glucose cotransporter 2 (SGLT2) inhibitors are medications with anti-inflammatory effects used to treat type 2 diabetes mellitus (T2DM). Cervical cancer is the most common gynecological cancer and is characterized by elevated inflammatory status. Accordingly, this study aimed to investigate the potential association between SGLT2 inhibitor use and cervical cancer development. In this retrospective cohort study, female patients with T2DM were divided into 2 groups: SGLT2 inhibitor users and a control group of non-SGLT2 inhibitor users. After propensity score matching, the SGLT2 inhibitor group and control group each had 136 212 patients. Cox proportional hazards regression was conducted to obtain the adjusted hazard ratio (aHR) and 95% confidence interval (CI) for cervical cancer between the 2 groups. Overall, 148 and 191 cases of cervical cancer were identified in the SGLT2 inhibitor and control groups, respectively. The incidence of cervical cancer was significantly lower in the SGLT2 inhibitor group than in the control group (aHR, 0.77; 95% CI, 0.62-0.96, *P* = 0.0179). In a subgroup analysis stratified by type of oral medication, the effect of SGLT2 inhibitors on cervical cancer development exhibited a significant difference compared with a biguanide group (aHR, 0.77; 95% CI, 0.63-0.95) and a sulfonylurea group (aHR, 0.69; 95% CI, 0.50-0.94) groups. In conclusion, the use of SGLT2 inhibitors in patients with T2DM is associated with reduced risk of cervical cancer development.

## Introduction

Type 2 diabetes mellitus (T2DM) is a common metabolic disease characterized by increased blood glucose levels 1. The primary pathophysiology for persistent hyperglycemia and subsequent T2DM involves the endogenous resistance of body cells to insulin stimulation 2. Treating T2DM includes the use of oral medications such as alpha-glucosidase inhibitors and biguanides, with insulin injections indicated for severe T2DM cases 3. In recent years, sodium-glucose cotransporter 2 (SGLT2) inhibitors have been widely used for T2DM management, reducing hyperglycemia by 0.5% to 1.0% 4, 5.

In addition to their anti-hyperglycemic function, SGLT2 inhibitors also protect other organs, as reported in previous publications 4, 6. Administration of SGLT2 inhibitors has been demonstrated to have neuroprotective effects and may be applied to treat cognitive impairment 7. The rate of myocardial infarction is considerably lower in T2DM individuals using SGLT2 inhibitors than in nonusers 8. Additionally, SGLT2 inhibitors have demonstrated protective effects on the kidneys, improving glomerular filtration rates 9. Regarding eye health, SGLT2 inhibitor use can reduce the risks of dry eye disease and diabetic retinopathy development 10-12.

Cervical cancer, a major cause of cancer-related mortality, primarily originates at the junction of the uterus and vagina 13-15. Known risk factors for cervical cancer include early age at first sexual intercourse, smoking, and previous human papillomavirus infection 16-18. However, few studies have evaluated the relationship between cervical cancer and SGLT2 inhibitor use in the T2DM population. Given that SGLT2 inhibitors can suppress inflammation, a pathophysiology of cervical cancer 19, 20, they may have a protective effect against cervical cancer formation.

This study investigated a potential correlation between SGLT2 inhibitor use and the incidence of cervical cancer. Data from Taiwan's National Health Insurance Research Database (NHIRD) were used for our analysis.

## Materials and Methods

### Data source

Our study adhered to the Declaration of Helsinki of 1964 and its subsequent amendments. In addition, this study was approved by the National Health Insurance Administration of Taiwan and the Institutional Review Board of Chung Shan Medical University Hospital (project code: CS1-20113). The requirement for written informed consent was waived by both institutions. Taiwan's NHIRD contains the claimed medical records of more than 23 million Taiwanese individuals for the period from January 1, 2000, to December 31, 2020. The data preserved in Taiwan's NHIRD are stratified by International Classification of Diseases-Ninth Revision (ICD-9) and International Classification of Diseases-Tenth Revision (ICD-10) diagnostic codes, age, sex, medical department codes, urbanization level, educational level, image examination codes, laboratory examination codes, procedure codes, and international ATC codes for medicine.

### Patient selection

**Figure 1:**
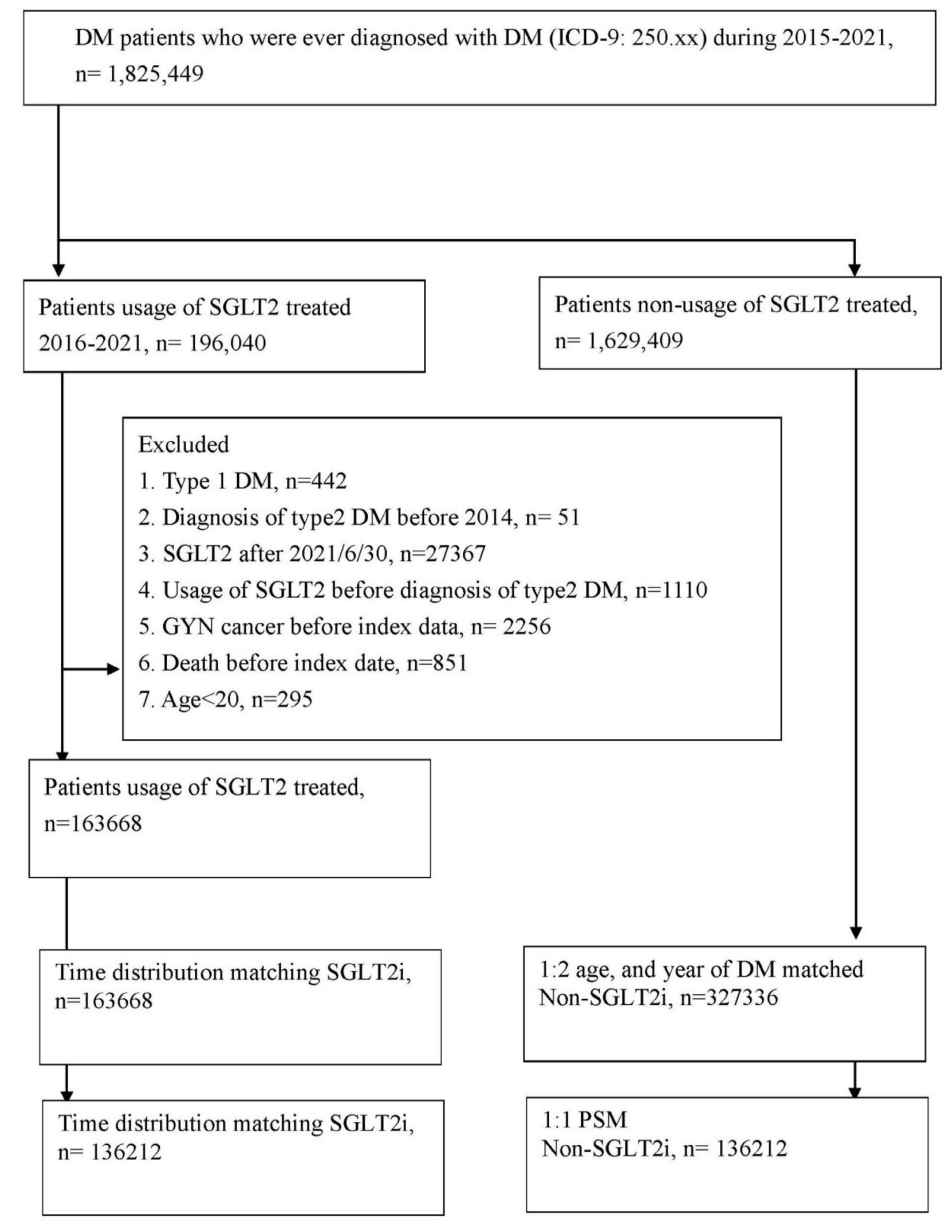
The flowchart of participant selection. NHIRD: National Health Insurance Research Database, N: number, PSM: propensity score-matching, SGLT2: sodium-glucose cotransporter 2.

This study was a retrospective cohort study. Patients with T2DM using SGLT2 inhibitors were defined on the basis of the following criteria: (1) the presence of ICD-9/ICD-10 codes indicating a T2DM diagnosis made between 2015 and 2021, (2) visits to an internal medicine doctor for more than 3 months, (3) the arrangement of glycated hemoglobin exams before T2DM diagnosis, and (4) the use of SGLT2 inhibitors such as dapagliflozin, canagliflozin, empagliflozin, or ertugliflozin as determined by ATC codes. The index date of our study was 6 months after the start of SGLT2 inhibitor use. The following exclusion criteria were applied to standardize our study population: (1) missing demographic data, (2) anti-hyperglycemic medicine use before T2DM diagnosis, (3) being aged below 20 years or above 100 years, and (4) the presence of gynecological cancers before the index date. Each patient with T2DM using SGLT2 inhibitors was matched to 2 T2DM participants who did not use SGLT2 inhibitors, who served as the control group. Propensity score matching (PSM) was applied to match the SGLT2 inhibitor group and the control group on the basis of demographics, comorbidities, and T2DM-related medication use. Consequently, 136 212 individuals were enrolled in the SGLT2 inhibitor group, and another 136 212 were enrolled in the control group. A flowchart illustrating the patient selection process is depicted in Figure 1.

### Primary outcome

The primary outcome of our study was the occurrence of cervical cancer as defined by the following criteria: (1) cervical cancer diagnosis based on ICD-9/ICD-10 diagnostic codes, (2) presence of pelvic exam procedure codes before or on the same day as the cervical cancer diagnosis, (3) occurrence of procedure codes indicating computed tomography or pelvic ultrasound exam before or on the same day as the cervical cancer diagnosis, and (4) diagnosis of cervical cancer by gynecologists.

### Covariate enrollment

Several covariates were considered in the present statistical analysis to adjust for their potential effects on the development of cervical cancer. These covariates were age, urbanization, hypertension, coronary heart disease, ischemic stroke, hyperlipidemia, hemorrhagic stroke, peripheral vascular disease, human papillomavirus infection, chronic kidney disease, diabetic retinopathy, and diabetic neuropathy, as diagnosed on the basis of ICD-9 or ICD-10 diagnostic codes. The number of comorbidities was integrated into the adjusted Diabetes Complications Severity Index (aDCSI) to assess T2DM severity. The use of various T2DM medications (as determined on the basis of ATC codes)—including alpha-glucosidase inhibitors, biguanides, sulfonylureas, thiazolidinediones, dipeptidyl peptidase-4 (DPP4) inhibitors, statins, and insulin—were included in the analysis. To ensure that the comorbidity and medication durations in the T2DM population were sufficiently long to influence cervical cancer risk, only comorbidities and medications present for more than 2 years before the index date in the NHIRD were considered in our analysis.

### Statistical analysis

Statistical analyses were conducted using SAS Statistics version 9.4 (SAS Institute, Cary, NC, USA). Descriptive analyses were employed to obtain the demographics, comorbidities, and T2DM medication distributions of the SGLT2 inhibitor and control groups. Absolute standardized differences (ASDs) were calculated to compare the parameters of the 2 groups, with an ASD value greater than 0.1 indicating a significant difference in our study. Cox proportional hazards regression was then performed to obtain adjusted hazard ratios (aHRs) with 95% confidence intervals (CIs) for cervical cancer incidence in the SGLT2 inhibitor group and the control group. This analysis was adjusted for the influence of demographics, comorbidities, and T2DM medication use. Statistical significance was set at *P* < 0.05.

## Results

Table 1 presents a comparison of basic features between the SGLT2 inhibitor group and the control group. The age distributions of the 2 groups were statistically identical (ASD < 0.1). Regarding comorbidities, the distributions of aDCSI scores were not significantly different between the 2 groups, likely because of the PSM procedure. Additionally, the rates of T2DM-related medication use—including use of sulfonylureas, alpha-glucosidase inhibitors, thiazolidinediones, DPP4 inhibitors, and insulin—were similar between the SGLT2 inhibitor group and the control group (all ASDs < 0.1; Table 1).

**Figure 2:**
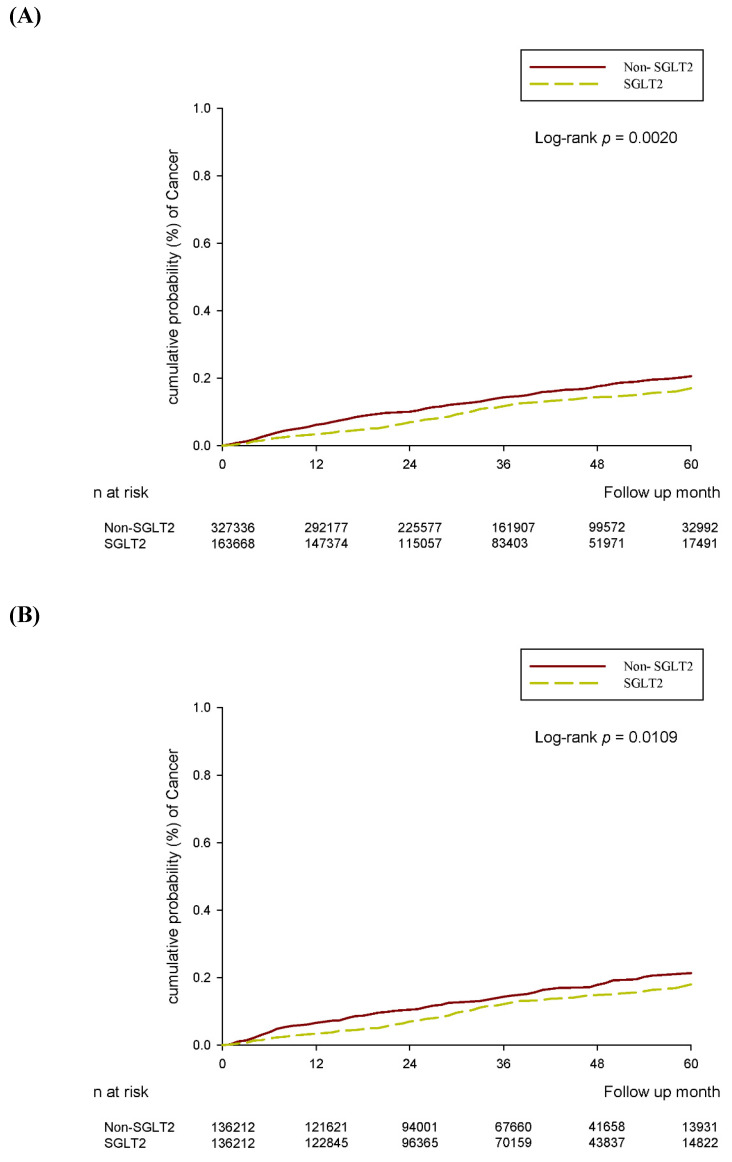
(A) 2:1 sex age matching. (B) After PSM.

After the follow-up period, 172 and 443 cervical cancer events were reported in the SGLT2 inhibitor group and control group, respectively, after 2:1 sex and age matching (aHR, 0.77; 95% CI, 0.63-0.93, *P* = 0.0081; Table 2). Through PSM, 148 and 191 cervical cancer episodes were revealed to have occurred in the SGLT2 inhibitor group and control group, respectively (Table 2). In the multivariable analysis, which adjusted for all covariates, the incidence of cervical cancer was significantly lower in the SGLT2 inhibitor group than in the control group (aHR, 0.77; 95% CI, 0.62-0.96, *P* = 0.0179; Tables 2 and 3). The cumulative probability of cervical cancer was lower in the SGLT2 inhibitor group than in the control group after both 2:1 sex and age matching (*P* = 0.0020) and PSM (*P* = 0.0109) (Figure 2). In the subgroup analysis stratified by oral medication, 159 and 264 events of cervical cancer were identified in the SGLT2 inhibitor group and the biguanide group, respectively (Table 4). The incidence of cervical cancer was significantly lower in the SGLT2 inhibitor group than in the biguanide group (aHR, 0.77; 95% CI, 0.63-0.95; Table 4). Furthermore, the effect of SGLT2 inhibitors on cervical cancer development exhibited a significant difference when compared with the sulfonylurea group (aHR, 0.69; 95% CI, 0.50-0.94; Table 4).

## Discussion

Our study demonstrated an association between the use of SGLT2 inhibitors and the risk of cervical cancer development. The use of SGLT2 inhibitors can reduce the likelihood of several morbidities 8, 9, 12. The most crucial mechanism of SGLT2 inhibitors is their anti-hyperglycemic function, which can reduce glycated hemoglobin levels by up to 1.0% 4. When used together with other anti-diabetic medications, the concurrent use of SGLT2 inhibitors and DPP4 inhibitors reduced the glycated hemoglobin level by 0.71% compared with DPP4 inhibitor monotherapy 21. Additionally, the use of SGLT2 inhibitors was demonstrated to reduce the inflammatory response in a model of autoimmune myocarditis 22. Specifically, SGLT2 inhibitors perform their anti-inflammatory function by downregulating the expression of several pro-inflammatory cytokines 6. In addition to inflammation suppression, SGLT2 inhibitors have antioxidant properties, which can reduce oxidative stress and related myocardium fibrosis 23. Furthermore, SGLT2 inhibitors can hinder the formation of reactive oxygen species in experimental diabetic kidney disease 19, and the antioxidant characteristic of SGLT2 inhibitors could alter the development of several diseases including liver diseases, neural defects, and neoplasm 24. If we focus on the correlation between SGLT2 inhibitors and cancers, both the SGLT2 inhibitors and malignancy precipitate euglycemic diabetic ketoacidosis while the interaction between SGLT2 inhibitors and cancer on euglycemic diabetic ketoacidosis risk remains unclear 25. Besides, previous studies demonstrated an inconclusive correlation between the risk of breast and bladder cancers and the usage of SGLT2 inhibitors 26, 27. On the other side, some preclinical researches exhibited that the SGLT2 inhibitors have anti-proliferative effects on several malignancies 28, and the possibilities of lung cancer and non-melanoma skin cancer may be reduced by SGLT2 inhibition 27. Moreover, the SGLT2 inhibitors were proven to trigger the apoptosis of cervical cancer call via modulating the sonic hedgehog signaling molecule expression in an experimental study 29. Regarding cervical cancer, inflammatory cytokines contribute to cervical cancer formation 20, and tumor-associated macrophages, altered under inflammatory conditions, facilitate cervical cancer metastasis 30. Oxidative stress is involved in the pathogenesis of cervical cancer 31, and antioxidants have potential for use in cervical cancer management 32. Additionally, hyperglycemic status can lead to cervical cancer development and poor prognosis for cervical cancer 33, 34. Because SGLT2 inhibitors can reduce pathways related to cervical cancer development 4, 19, their use in the T2DM population may correlate with a lower rate of cervical cancer. Our study's results support this opinion.

In our study, the use of SGLT2 inhibitors in the T2DM population was associated with a reduced likelihood of cervical cancer occurrence. Previous studies have indicated that SGLT2 inhibitors can reduce the proliferation of certain malignancies, including breast and liver cancer cells 35, 36. Regarding the correlation between cervical cancer and SGLT2 inhibitors, the previous experimental research and bioinformatic analysis demonstrated that the application of SGLT2 inhibitors can reduce the cervical cancer cell growth and migration 27-29, 37, 38. Nevertheless, the association between SGLT2 inhibitors utilization and the incidence of subsequent cervical cancer in real world had not been reported. To our knowledge, our results could be a preliminary experience that revealed the negative correlation between SGLT2 inhibitors utilization and consecutive cervical cancer occurrence in patients with T2DM clinically. Comparing to previous experimental studies 27-29, 37, 38, we expanded their results and exhibited that the SGLT2 inhibitor may indeed related to the lower risk of cervical cancer in real world, which is the novelty of our study. In our study, patients with T2DM diagnosed with cervical cancer within 6 months of SGLT2 inhibitor use were excluded to ensure a clear temporal relationship between SGLT2 inhibitor use and the occurrence of cervical cancer. Furthermore, established risk factors such as human papillomavirus infection and T2DM severity were adjusted for in our multivariable regression analysis to account for these confounders 15, 16, 34. Correspondingly, SGLT2 inhibitor use may be an independent protective factor against cervical cancer development. The mechanism by which SGLT2 inhibitors suppress liver cancer cells involves the regulation of inflammatory cytokines such as interleukin-8 and tissue inhibitors of metalloproteinase-1 36. Therefore, the relationship between SGLT2 inhibitor use and reduced incidence of cervical cancer may be primarily due to its anti-inflammatory effects. Further studies are required to clarify this concept and to explore the underlying mechanisms.

T2DM is a common metabolic disease affecting more than 10% of the global population 3. Additionally, the prevalence of T2DM is increasing, with projections suggesting that approximately 700 million individuals will be diagnosed with T2DM by 2040 2. SGLT2 inhibitors have demonstrated their efficacy in reducing serum glucose levels, and they are frequently used for T2DM management 3, 39. In the United States, more than 10% of patients with T2DM use SGLT2 inhibitors to reduce their serum glucose concentration 40. Cervical cancer is the third-to-fourth leading malignancy among women following breast cancer and colorectal cancer 41, 42. In 2018, approximately 570 000 cases of cervical cancer were diagnosed, resulting in 311 000 deaths 13. Given the considerable prevalence of both SGLT2 inhibitor use and cervical cancer 39, 43, investigating the potential association between them is crucial.

Our study had several limitations. First, the NHIRD is a claims database that does not contain actual medical records. Consequently, crucial information—such as the serum glucose levels and glycated hemoglobin concentrations of patients with T2DM, the severity of T2DM-related complications, treatment responses after SGLT2 inhibitor use, details of other comorbidities such as human papillomavirus infection and sexual intercourse, actual sites of cervical cancer, imaging results related to cervical cancer, pathological reports of cervical cancer, responses to treatment for cervical cancer, and recurrence of cervical cancer (if any)—was not considered in our study. Second, the retrospective design of our study might have contributed to low homogeneity between the groups despite the application of PSM. Additionally, although smoking is a well-known risk factor for cervical cancer development 41, we could not include this covariate in our analysis because the related code is rarely entered by physicians in clinical practice. Similarly, the use of oral contraceptives, another risk factor for cervical cancer formation 15, was not included in our research because such contraceptives are self-paid in Taiwan.

In conclusion, the use of SGLT2 inhibitors in patients with T2DM correlated with a significantly reduced incidence of subsequent cervical cancer after adjustment for multiple covariates. Accordingly, SGLT2 inhibitors can be recommended for patients with both T2DM and preexisting risk factors for cervical cancer. In the field of cancer intervention, the usage of SGLT2 inhibitors might be applied as a new preventive or adjuvant intervention for the T2DM population with precancerous cervix conditions or cervical cancer. Further large-scale prospective clinical trial to evaluate the exact role of SGLT2 inhibitors on cervical cancer in real world and the association between SGLT2 inhibitors utilization and the treatment outcome of cervical cancer is mandatory.

## Figures and Tables

**Table 1:** Baseline characteristics of the features in Non-SGLT2 inhibitors user and SGLT-2 inhibitor user.

	2:1 sex age matching			After PSM		
	Non- SGLT2	SGLT2	ASD	Non- SGLT2	SGLT2	ASD
N	327336	163668		136212	136212	
Age			0.0000			0.0679
20-39	17670 (5.40%)	8835 (5.40%)		4249 (3.12%)	6028 (4.43%)	
40-49	36722 (11.22%)	18361 (11.22%)		13266 (9.74%)	14401 (10.57%)	
50-59	80116 (24.48%)	40058 (24.48%)		33503 (24.60%)	33603 (24.67%)	
60-69	111622 (34.10%)	55811 (34.10%)		49297 (36.19%)	47772 (35.07%)	
70-79	60504 (18.48%)	30252 (18.48%)		27020 (19.84%)	25898 (19.01%)	
>=80	20702 (6.32%)	10351 (6.32%)		8877 (6.52%)	8510 (6.25%)	
Urbanization			0.0453			0.0370
Urban	191091 (58.38%)	98397 (60.12%)		79727 (58.53%)	80828 (59.34%)	
Sub-urban	103456 (31.61%)	50052 (30.58%)		43112 (31.65%)	42377 (31.11%)	
Rural	32789 (10.02%)	15219 (9.30%)		13373 (9.82%)	13007 (9.55%)	
aDCSI score			0.2851			0.0000
0	200570 (61.27%)	77452 (47.32%)		67685 (49.69%)	67559 (49.60%)	
1-2	101960 (31.15%)	69994 (42.77%)		56413 (41.42%)	56284 (41.32%)	
>=3	24806 (7.58%)	16222 (9.91%)		12114 (8.89%)	12369 (9.08%)	
Comorbidities						
Hypertension	169611 (51.82%)	101028 (61.73%)	0.2011	84407 (61.97%)	83114 (61.02%)	0.0195
CAD	30924 (9.45%)	23051 (14.08%)	0.1443	16787 (12.32%)	17363 (12.75%)	0.0128
Hyperlipidemia	167061 (51.04%)	108528 (66.31%)	0.3140	89765 (65.90%)	88625 (65.06%)	0.0176
Ischemic stroke	13958 (4.26%)	7799 (4.77%)	0.0241	6535 (4.80%)	6391 (4.69%)	0.0050
Hemorrhage stroke	2289 (0.70%)	1164 (0.71%)	0.0014	927 (0.68%)	904 (0.66%)	0.0021
Kidney disease	32156 (9.82%)	16497 (10.08%)	0.0086	13266 (9.74%)	13354 (9.80%)	0.0022
Rheumatoid arthritis	3557 (1.09%)	1449 (0.89%)	0.0204	1286 (0.94%)	1270 (0.93%)	0.0012
Systemic lupus erythematosus	736 (0.22%)	252 (0.15%)	0.0163	207 (0.15%)	211 (0.15%)	0.0008
Sicca/Sjogren syndrome	3856 (1.18%)	1471 (0.90%)	0.0276	1343 (0.99%)	1282 (0.94%)	0.0046
Ankylosing spondylitis	2489 (0.76%)	1185 (0.72%)	0.0042	1005 (0.74%)	984 (0.72%)	0.0018
COPD	8539 (2.61%)	4241 (2.59%)	0.0011	3460 (2.54%)	3461 (2.54%)	0.0001
Medication						
NSAIDs	194966 (59.56%)	99533 (60.81%)	0.0256	82456 (60.54%)	82479 (60.55%)	0.0004
Corticosteroids	65684 (20.07%)	34487 (21.07%)	0.0249	28065 (20.60%)	28109 (20.64%)	0.0008
PPI	28343 (8.66%)	15088 (9.22%)	0.0196	11822 (8.68%)	12035 (8.84%)	0.0055
Aspirin	55706 (17.02%)	38670 (23.63%)	0.1648	30556 (22.43%)	30661 (22.51%)	0.0019
Statin	161792 (49.43%)	119710 (73.14%)	0.5020	96479 (70.83%)	96160 (70.60%)	0.0052
Alpha-blockers	5612 (1.71%)	2996 (1.83%)	0.0088	2420 (1.78%)	2408 (1.77%)	0.0007
Beta- blockers	86740 (26.50%)	54864 (33.52%)	0.1537	43319 (31.80%)	43556 (31.98%)	0.0037
CCBs	87183 (26.63%)	44789 (27.37%)	0.0165	38064 (27.94%)	37294 (27.38%)	0.0126
ACEI	13903 (4.25%)	9126 (5.58%)	0.0615	7152 (5.25%)	7221 (5.30%)	0.0023
ARBs	132465 (40.47%)	91676 (56.01%)	0.3149	74360 (54.59%)	73926 (54.27%)	0.0064
Biguanides	179428 (54.81%)	149456 (91.32%)	0.9028	124185 (91.17%)	122073 (89.62%)	0.0526
Sulfonylureas	73873 (22.57%)	66089 (40.38%)	0.3908	51793 (38.02%)	51245 (37.62%)	0.0083
Alpha glucosidase inhibitors	22845 (6.98%)	27730 (16.94%)	0.3107	17947 (13.18%)	19291 (14.16%)	0.0287
Thiazolidinediones	23704 (7.24%)	29103 (17.78%)	0.3227	19725 (14.48%)	20718 (15.21%)	0.0205
DPP4	54086 (16.52%)	62791 (38.36%)	0.5048	41485 (30.46%)	44649 (32.78%)	0.0500
Insullin	38605 (11.79%)	39236 (23.97%)	0.3219	25876 (19.00%)	27684 (20.32%)	0.0334
GLP-1	3215 (0.98%)	3566 (2.18%)	0.0961	2587 (1.90%)	2666 (1.96%)	0.0042

COPD: chronic obstructive pulmonary disease, CAD: Coronary Artery Disease, GLP-1: Glucagon-like peptide-1 ASD: absolute standardized difference, PSM: propensity score matching.

**Table 2:** Incidence rate of cervical cancer in Non-SGLT-2 inhibitors user and control groups

	2:1 sex age matching			After PSM		
	Non- SGLT2	SGLT2	P value	Non- SGLT2	SGLT2	P value
N	327336	163668		136212	136212	
Follow up person months	11192382	5706217		4668288	4777101	
New case	443	172		191	148	
Incidence rate* (95% C.I.)	0.40 (0.36-0.43)	0.30 (0.26-0.35)		0.41 (0.36-0.47)	0.31 (0.26-0.36)	
Crude Relative risk (95% C.I.)	reference	0.76 (0.64-0.91)	0.0027	reference	0.76 (0.61-0.94)	0.0119
Adjusted HR* (95% C.I.)†	reference	0.77 (0.63-0.93)	0.0081	reference	0.77 (0.62-0.96)	0.0179

*Incidence rate, per 10,000 person-months † adjusted hazard ratio, the covariates including year of index, sex, age, Urbanization, Insurance property, aDCSI score, co-morbidities, and medication at baseline.

**Table 3:** Multiple Cox regression to estimate the hazard ratio in this study.

	aHR(95% CI )	
	2:1 sex age matching	After PSM
Study		
Non-SGLT2	reference	reference
SGLT2	0.77 (0.63-0.93)	0.77 (0.62-0.96)
Age		
20-39	reference	reference
40-49	3.65 (1.74-7.63)	5.40 (1.29-22.66)
50-59	3.93 (1.92-8.04)	6.48 (1.59-26.45)
60-69	4.25 (2.08-8.68)	6.85 (1.68-27.92)
70-79	4.44 (2.14-9.22)	7.69 (1.86-31.75)
>=80	5.98 (2.78-12.85)	7.19 (1.65-31.29)
Comorbidity (ref: non)		
Hypertension	1.12 (0.91-1.38)	1.04 (0.79-1.37)
CAD	0.94 (0.70-1.26)	0.86 (0.59-1.25)
Hyperlipidemia	1.00 (0.83-1.20)	1.06 (0.83-1.35)
Ischemic stroke	1.09 (0.74-1.61)	1.19 (0.72-1.97)
Hemorrhage stroke	1.67 (0.82-3.42)	1.73 (0.62-4.77)
Kidney disease	1.26 (0.94-1.68)	1.56 (1.09-2.24)
Rheumatoid arthritis	1.31 (0.65-2.67)	2.26 (1.00-5.12)
Sicca/Sjogren syndrome	1.61 (0.85-3.03)	0.61 (0.15-2.47)
Ankylosing spondylitis	0.69 (0.22-2.15)	0.43 (0.06-3.07)
COPD	0.93 (0.56-1.54)	1.05 (0.54-2.06)

**Table 4:** Incidence rate of cervical cancer by oral medications.

	2:1 sex age matching	
	Biguanides	SGLT2
N	179428	149456
Follow up person months	6351688	5262900
New case	264	159
Incidence rate*(95% C.I.)	0.42 (0.37-0.47)	0.30 (0.26-0.35)
Crude Relative risk (95% C.I.)	reference	0.73 (0.60-0.88)
Adjusted HR* (95% C.I.)†	reference	0.77 (0.63-0.95)
	Sulfonylureas	SGLT2
N	73873	66089
Follow up person months	2701750	2347102
New case	122	67
Incidence rate*(95% C.I.)	0.45 (0.38-0.54)	0.29 (0.22-0.36)
Crude Relative risk (95% C.I.)	reference	0.63 (0.47-0.85)
Adjusted HR* (95% C.I.)†	reference	0.69 (0.50-0.94)
